# eQTL mapping of rare variant associations using RNA-seq data: An evaluation of approaches

**DOI:** 10.1371/journal.pone.0223273

**Published:** 2019-10-03

**Authors:** Sharon Marie Lutz, Annie Thwing, Tasha Fingerlin

**Affiliations:** 1 Department of Population Medicine, Harvard Medical School and Harvard Pilgrim Health Care, Boston, MA, United States of America; 2 Department of Biostatistics, Harvard T.H. Chan School of Public Health, Boston, MA, United States of America; 3 Department of Biostatistics and Informatics, University of Colorado, Anschutz Medical Campus, Aurora, CO, United States of America; 4 Center for Genes, Environment, and Health, National Jewish Health, Denver, CO, United States of America; University of North Dakota, UNITED STATES

## Abstract

Expression quantitative trait loci (eQTL) provide insight on transcription regulation and illuminate the molecular basis of phenotypic outcomes. High-throughput RNA sequencing (RNA-seq) is becoming a popular technique to measure gene expression abundance. Traditional eQTL mapping methods for microarray expression data often assume the expression data follow a normal distribution. As a result, for RNA-seq data, total read count measurements can be normalized by normal quantile transformation in order to fit the data using a linear regression. Other approaches model the total read counts using a negative binomial regression. While these methods work well for common variants (minor allele frequencies > 5% or 1%), an extension of existing methodology is needed to accommodate a collection of rare variants in RNA-seq data. Here, we examine 2 approaches that are direct applications of existing methodology and apply these approaches to RNAseq studies: 1) collapsing the rare variants in the region and using either negative binomial regression or Poisson regression and 2) using the normalized read counts with the Sequence Kernel Association Test (SKAT), the burden test for SKAT (SKAT-Burden), or an optimal combination of these two tests (SKAT-O). We evaluated these approaches via simulation studies under numerous scenarios and applied these approaches to the 1,000 Genomes Project.

## Introduction

Expression quantitative trait loci (eQTL) studies provide insight on transcription regulation and have the potential to illuminate the molecular basis of phenotypic outcomes. [1] As a result, high-throughput RNA sequencing (RNA-seq) is gaining in popularity as a technique to measure gene expression abundance. [2], [3] RNA-seq offers several advantages over microarrays, such as being less noisy, having a much larger dynamic range, having the potential to identify new transcripts, and being able to measure allele-specific expression (ASE). [3]

Traditional eQTL mapping methods for microarray expression data often assume the expression data follow a normal distribution and involve application of linear regression or equivalent approaches for eQTL mapping. [4] To meet the assumptions of a linear regression for RNA-seq data, total read count measurements can be normalized by normal quantile transformation. [5] Given the inherent count nature of the sequencing read data, instead of transforming the total read count measurements, a negative binomial or Poisson regression can be used. [6], [7], [8] For small sizes with multiple replications per subject, methods such as edgeR use a negative binomial regression with the dispersion parameter calculated from the multiple replications. [9], [10]

These methods for eQTL mapping in RNA-seq data readily accommodate common variants (minor allele frequency (MAF) > 5%), but were not specifically designed for a collection of rare variants. Several methods have been proposed for testing the association between sequence data with rare variants and a normally distributed or dichotomous outcome. Most methods for analyzing rare variants fall into two categories: burden tests and variance-based tests. Burden tests collapse rare variants within a region and use an indicator function, sum, or proportion as the genetic variable in an association test. [11], [12] Most of these tests assume that the rare variants influence the phenotype in the same direction, and collapsing across all variants is likely to introduce noise since most rare and common variants have little or no effect on the outcome of interest. One of the most popular variance-based tests is the sequence kernel association test (SKAT). [12] SKAT is a regression approach that tests for association between variants in a region (potentially both common and rare) and a dichotomous or continuous, normally-distributed phenotype while adjusting for covariates. [12] SKAT optimal (SKAT-O) combines the variance-based test SKAT with a burden approach. [13] Numerous extensions of SKAT have been proposed and applied, such as an application of SKAT to a normalized outcome using inverse normal transformation to analyze rare variants. [14]

While these methods are used in a wide variety of settings, most of the association methods for rare variants are for normally distributed or dichotomous phenotypes rather than count data like RNA-seq data. Here, we examine 2 extensions of existing methodology to analyze rare variants in RNA-seq data: 1) collapsing the rare variants in the region and using either negative binomial regression or Poisson regression and 2) using the normalized read counts with the Sequence Kernel Association Test (SKAT), the burden test for SKAT (SKAT-Burden), or an optimal combination of these two tests (SKAT-O). We evaluated these 2 approaches via simulation studies under numerous scenarios. We then applied these approaches to the 1000 Genomes Project Consortium data to determine if the genes that show strong differentiation between closely related populations are acting on expression of nearby genes.

## Materials and methods

Consider a collection of *J* rare variants in a gene indexed by *j* = 1, …, *J*. Let *x*_*ij*_ = 0, 1, and 2 for 0, 1, and 2 copies of the disease allele, respectively, for subject *i* and SNP *j*. Let *t*_*i*_ be the total number of reads mapped to this gene for subject *i* and *y*_*i*_ be the normalized read count. Let *C*_*i*_ be a vector of *k* covariates for subject *i*, where *i* = 1,.., *n*.

### Approach 1: Collapsing the rare variants in the region and using negative binomial regression or Poisson regression

We consider modeling the total number of reads *t*_*i*_ by a Poisson distribution or a negative binomial distribution, depending on whether there is significant over dispersion for the Poisson distribution. [15] The density function for a negative binomial distribution is the following:

$$\begin{array}{c} f_{NB}\left(t_{i}|\mu_{i},\phi \right)=\frac{\Gamma (t_{i}+1/\phi )}{t_{i}!\Gamma \left(1/\phi \right)}{\left(\frac{1}{1+\phi \mu_{i}}\right)}^{1/\phi }{\left(\frac{\phi \mu_{i}}{1+\phi \mu_{i}}\right)}^{t_{i}} \end{array}$$ (1)

with mean *μ*_*i*_, dispersion parameter *ϕ*, and a covariate vector *C*_*i*_ = (*c*_*i*1_, …, *c*_*iK*_)^*T*^. When applying either a Poisson or negative binomial regression, one can employ a log link function to acknowledge the fact that *μ*_*i*_ > 0:

$$\begin{array}{c} log\left(\mu_{i}\right)=\alpha_{0}+\alpha_{k}k_{i}+\alpha_{c}^{T}C_{i}+\alpha_{x}f\left(x_{i}\right) \end{array}$$ (2)

where *k*_*i*_ is the total number of reads mapped to a given gene for subject *i* and *f*(*x*_*i*_) is a function of the rare variants in the region, either $f\left(x_{i}\right)=\sum_{j=1}^{J}x_{ij}$ (i.e. the sum of rare variants within a predefined region such as a gene) or $f\left(x_{i}\right)=I_{x_{ij}>0}$ (i.e. an indicator that equals 1 if subject *i* has at least one rare variant present in the region).

The null and alternative hypothesis to test for an association between the read counts and the collapsed rare variants in the region can be written as follows:

$$\begin{array}{c} \begin{array}{c} H_{0}:\alpha_{x}=0\\ H_{1}:\alpha_{x}\neq 0 \end{array} \end{array}$$ (3)

Parameter estimation can be obtained through maximum likelihood estimation (MLE). Since there is not a closed form for the MLEs, iterative techniques such as the Newton-Raphson algorithm can be used. Hypothesis testing can be done using Wald, score, or likelihood ratio tests.

The advantage of Approach 1 is that it is easy to implement. Poisson and negative binomial regressions are usually faster to run than SKAT. The disadvantage of Approach 1 is that taking the sum of the rare variants in the region or using an indicator function for at least one rare variant in the region assumes that all of the rare variants influence the phenotype in the same direction. [12] Collapsing across all variants may also introduce noise. Also, if there is over dispersion, the Poisson regression may have an inflated type 1 error rate.

### Approach 2: Using the normalized read counts with SKAT, SKAT-O, or SKAT-Burden

For the normalized read count *y*_*i*_, a genotype vector *X*_*i*_ = (*x*_*i*1_, …, *x*_*iJ*_)^*T*^, and a covariate vector *C*_*i*_ = (*c*_*i*1_, …, *c*_*iK*_)^*T*^, then

$$\begin{array}{c} y_{i}=\alpha_{0}+\alpha_{c}^{T}C_{i}+\alpha_{x}^{T}X_{i}+\epsilon_{i} \end{array}$$ (4)

where the error term *ϵ*_*i*_ ∼ *N*(0, *σ*^2^) and $\alpha_{x}=\left(\alpha_{x_{1}},...,\alpha_{x_{J}}\right)$.

The null and alternative hypothesis to test for an association between the transformed read counts and the rare variants in the region can be written as follows:

$$\begin{array}{c} \begin{array}{c} H_{0}:\alpha_{x}=0\\ H_{1}:\alpha_{x}\neq 0 \end{array} \end{array}$$ (5)

In order to increase the power to test the null hypothesis, SKAT assumes that $\alpha_{x_{j}}$ comes from an arbitrary distribution with mean $\mu_{\alpha_{x_{j}}}=0$ and variance $\sigma_{\alpha_{x_{j}}}^{2}=w_{j}\tau$ where *w*_*j*_ is a pre-specified weight for variant *j*. As a result, testing the null hypothesis *H*_0_: *α*_*x*_ = 0 is equivalent to testing the null hypothesis *H*_0_: *τ* = 0, which can be performed with a variance-component score statistic

$$\begin{array}{c} Q={\left(y-\hat{\mu }\right)}^{T}K\left(y-\hat{\mu }\right) \end{array}$$ (6)

where $\hat{\mu }={\hat{\alpha }}_{0}+{\hat{\alpha_{C}}}^{T}C$ is the predicted mean of y under the null hypothesis. *K* = *XWX*^*T*^ where *K*() is the weighted linear kernel function and *W* = *diag*(*w*_1_, …, *w*_*p*_) is a weight matrix for the *J* variants. Under the null, Q follows a mixture of chi-square distributions, which can be approximated using the Davies method. [12] [16]

The choice of the weights plays an important role in SKAT where good choices of the weights can improve power. [12] If the weight for variant *j*, *w*_*j*_, is close to zero, then variant *j* makes only a small contribution to variance-component score statistic *Q*. It is recommended to set the weight for variant *j* from a beta distribution such that $\sqrt{w_{j}}=Beta\left(MAF_{j};a_{1};a_{2}\right)$ where MAF_*j*_ is the sample minor-allele frequency for variant *j* in the data. The prespecified parameters *a*_1_ and *a*_2_ can vary. For example, to allow rare variants to have a larger effect, one can set 0 < *a*_1_ ≤ 1 and *a*_2_ ≥ 1. The default for the SKAT software in R is to set *a*_1_ = 1 and *a*_2_ = 25 because this increases the weight of rare variants while still putting nonzero weights for uncommon variants with MAF 1%–5%. [12] These default parameters are the values that we used for all simulations.

While the above describes the basis of SKAT, the method has been extended to include a burden test (SKAT-burden) and an optimal test (SKAT-O) that combines the burden test and the traditional version of SKAT. [13] We considered all three of these methods (SKAT, SKAT-burden, and SKAT-O) using an inverse normal transformation for the read counts.

The advantage of Approach 2 is that it avoids introducing noise by collapsing the rare variants in the region. Approach 2 is more likely to correctly model the rare variants than Approach 1. The disadvantage of Approach 2 is that by using the normalized read counts information may be lost in terms of the outcome.

### Simulations

Using the software package SKAT, 1,000 rare variant datasets were generated from a 3kb region where 10% markers with MAF < 0.005 were generated as causal for 15, 30, 50, 100, and 500 subjects, respectively. There were 58 rare and common variants in the region. We filtered out all common variants (MAF > 5%), which resulted in 54 variants in the region. We considered the following 2 scenarios to simulate the total read counts for each subject for a given gene.

#### Scenario A

Total read counts were generated from a negative binomial distribution or a Poisson distribution such that

**Scenario A.1** *t*_*i*_ ∼ *Poisson*(*μ* * (1 − *I*_*RV*_) + (*fold* * *μ*) * *I*_*RV*_)

**Scenario A.2** *t*_*i*_ ∼ *NegativeBinomial*(*μ* * (1 − *I*_*RV*_) + (*fold* * *μ*) * *I*_*RV*_, *ϕ*)

where *I*_*RV*_ is an indicator that equals one if the subject has any causal variants within the region, the average total read count *μ* = 50, 100, 500, the dispersion parameter *ϕ* = 1.1 and fold change varies from 1 to 2 by 0.1 and 3, 4, 8.

#### Scenario B

We also considered the number of causal variants within the region such that

**Scenario B.1** If a subject has no causal variants in the region then *t*_*i*_ ∼ *Poisson*(*μ*). If that subject has any causal variants in the region *t*_*i*_ ∼ *Poisson*(*fold* * *μ* * *n*_*cv*_)

**Scenario B.2** If a subject has no causal variants in the region then *t*_*i*_ ∼ *NegativeBinomial*(*μ*, *ϕ*). If that subject has any causal variants in the region *t*_*i*_ ∼ *NegativeBinomial*(*fold* * *μ* * *n*_*cv*_, *ϕ*)

where *n*_*cv*_ is the number of causal variants in the region, *μ* = 50, 100, 500, the dispersion parameter *ϕ* = 1.1 and fold change varies from 1 to 2 by 0.1 and 3, 4, 8.

## Results and discussion

**Fig 1:**
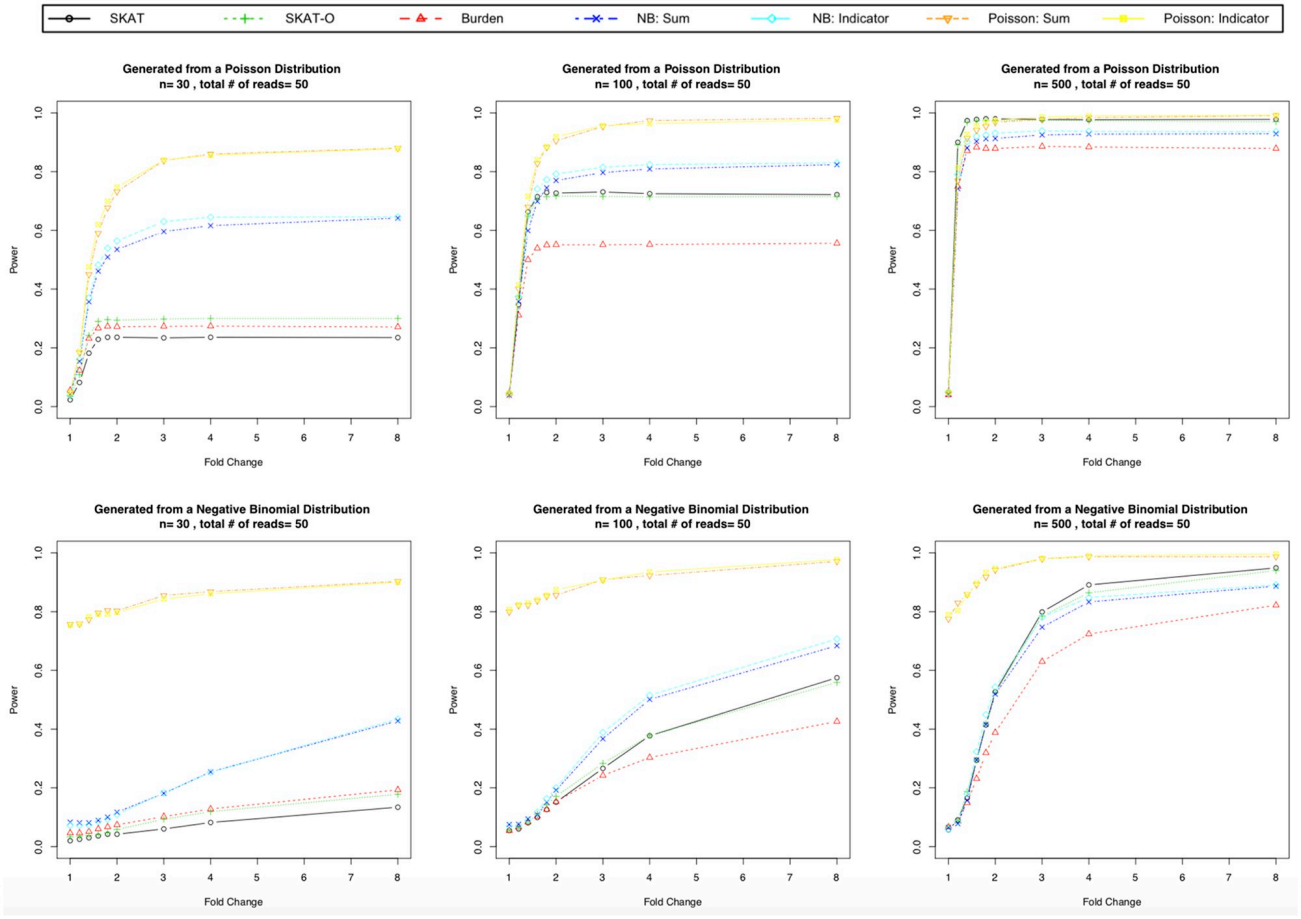
Simulation results for Scenario A. The read counts were generated from a Poisson distribution for row 1 and a negative binomial distribution for row 2. All plots were generated from Scenario A with the average number of reads *μ* = 50. Columns 1-3 are for 30, 100, and 500 subjects, respectively. For data generated under the Poisson distribution (row 1), all methods preserved the type 1 error rate (Fold Change = 1) and the Poisson regressions had substantial gains in power over the other methods for smaller sample sizes (*n* = 30, 100). For data generated under the negative binomial distribution (row 2), the Poisson regressions had an inflated type 1 error rate (Fold Change = 1). For a sample size of 500, SKAT and SKAT-O had the highest power.

Results for all 60 plots of the 540 simulation scenarios considered (i.e. 9 fold changes for all combinations of *μ* = 50, 100, 500 and *n* = 15, 30, 50, 100, 500 subjects for scenarios A.1, A.2, B.1, B.2) are given in the S1 File. The simulation results for scenario A were similar to scenario B, so we only show the results for scenario A here, but include the results for scenario B in the S1 File. Fig 1 shows the power and type 1 error rates (fold Change = 1) for Scenarios A.1 and A.2 for *n* = 30, 100, 500 and the average number of reads *μ* = 50.

### Results: Poisson distribution

As seen in Fig 1, when the underlying distribution of read counts is Poisson, all proposed methods preserved the type 1 error rate (i.e. fold Change = 1). The Poisson regression using the sum of the rare variants or the indicator of the presence or absence of rare variants had substantially higher power compared to the other methods for the smallest sample size (i.e *n* = 30). For the largest sample size we considered (*n* = 500) under scenario A.1, SKAT and SKAT-O using the normalized read count and the Possion regressions had the most power.

### Results: Negative binomial distribution

As seen in Fig 1, when the underlying distribution of read counts is negative binomial, the Poisson regressions had an inflated type 1 error rate. This is expected since the Poisson regressions ignore the dispersion. For *n* = 15, 30, the negative binomial regressions had an inflated type 1 error. For *n* = 500, SKAT and SKAT-O using the normalized read counts had the highest power. Given that the SKAT methods (SKAT, SKAT-O, and SKAT-Burden) using the normalized read counts were the only methods that maintained the type 1 error rate in all scenarios, we recommend these methods over the other approaches.

### Data analysis

We applied these proposed approaches to the 1000 Genomes Project Consortium publicly available data [17]. This study on the global reference for human genetic variation found four genes that showed strong differentiation between closely related populations, which highlights the rarity of strong selective sweeps in recent human evolution. [17] The four genes are as follows:

1. LCT [chromosome 2] associated with lactose tolerance
2. FADS cluster [chromosome 11] that may be associated with dietary fat
3. SLC24A5 [chromosome 15] associated with skin pigmentation
4. HERC2 [chromosome 15] associated with eye color

The study also found several potentially novel selection signals including:

1. TRBV9 [chromosome 7]
2. PRICKLE4 [chromosome 6]

Given these results, we wanted to determine if rare variants within these 6 genes demonstrated association with expression of the respective gene. There was very sparse coverage of the *TRBV9* gene and we excluded this gene from our analyses. Using the total read counts for 87 subjects with African ancestry from Yoruba and 266 subjects with European ancestry from England, Scotland, Italy, and Utah residents with Northern and Western European ancestry, we applied the above approahces to determine if the rare variants in these genes are associated with the overall expression levels for these genes in these 2 populations.

As seen in Table 1, the rare variants in the *LCT* region on chromosome 2 were significantly associated with overall expression in the subjects with African Ancestry using the SKAT-Burden (p-value = 5.2E-4) and SKAT-O (p-value = 1.1E-3) approaches. This same region was marginally associated in the European Ancestry group using the SKAT-Burden (p-value = 0.03) and SKAT-O (p-value = 0.06) approaches. Based on the simulation studies, the SKAT methods (SKAT, SKAT-O, and SKAT-Burden) using the normalized read counts were the only methods that maintained the type 1 error rate in all scenarios and were thus the recommended approaches. These proposed methods were able to detect an association of the rare variants in the *LCT* region with overall expression. However, since both groups had a relatively small sample size (n = 87 for the subjects with African ancestry and n = 266 for the subjects with European ancestry), further study of this region is needed.

**Table 1:** Using the total read counts for each of the 87 Yoruba subjects with African Ancestry and 266 subjects with European ancestry from the 1000 genomes project, we applied the 7 approaches to determine if the rare variants in these genes are associated with the overall expression levels for these genes. Below are the p-values for all approaches and 5 genes (*LCT*, *PRICKLE4*, *FADS*, *SLC24A5*, *HERC2*). Sum refers to the sum of the rare variants in the region and Indicator refers to the indicator function which equals one if the subject has at least one rare variant in the region.

		*LCT*	*PRICKLE4*	*FADS*	*SLC24A5*	*HERC2*
Population	Method	Chr 2	Chr 6	Chr 11	Chr 15	Chr 15
African Ancestry	SKAT-Burden	**5.2E-4**	0.30	0.04	0.68	0.22
	SKAT-O	**1.1E-3**	0.46	0.07	0.48	0.34
	SKAT	0.04	0.74	0.15	0.30	0.27
	Negative Binomial: Sum	0.04	0.55	0.01	0.52	0.25
	Negative Binomial: Indicator	0.52	0.37	0.08	0.52	0.57
	Poisson: Sum	2.2E-19	1.0E-42	0	3.5E-08	2.2E-198
	Poisson: Indicator	0.01	2.1E-100	0	3.0E-07	1.4E-34
European Ancestry	SKAT-Burden	**0.03**	0.51	0.51	0.77	0.50
	SKAT-O	**0.06**	0.56	0.60	1.00	0.73
	SKAT	0.18	0.43	0.39	0.97	0.57
	Negative Binomial: Sum	0.12	0.62	0.38	0.62	0.63
	Negative Binomial: Indicator	0.41	0.62	0.41	0.21	0.70
	Poisson: Sum	1.5E-19	1.4E-38	1.9E-164	8.5E-05	1.2E-40
	Poisson: Indicator	4.7E-05	1.4E-38	1.5E-141	4.5E-19	1.9E-23

None of the other regions (*PRICKLE4, FADS, SLC24A5, HERC2*) were significantly associated with the overall expression using any method other than the Poisson regressions. Both of the Poisson regression approaches had an inflated type 1 error in Scenarios A.2 and B.2 in our simulation studies and were, therefore, not recommended. These analyses demonstrate the potential for substantially disparate conclusions depending on the method used and highlight the advantage of using the SKAT methods with normalized read counts to detect eQTLs of rare variant associations in RNA-seq data.

## Conclusions

It has been previously shown that an increase in power can be achieved for eQTL mapping with RNA-seq data by using a negative binomial regression with the total read count instead of normalizing the read count and using a linear regression. [6] While this method works well for common variants, in the scenarios we considered with rare variants, either the read counts were transformed or the rare variants within the region were collapsed.

Based on the simulation studies that were performed, the SKAT (SKAT, SKAT-O, and SKAT-Burden) methods using the normalized read counts were the only methods that maintained the type 1 error rate in all scenarios for all sample sizes. Therefore, we recommend using SKAT with normalized read counts over the other approaches considered here. The data analysis further supported this recommendation. The 2 methods that found a significant association of rare variants in the *LCT* region on chromosome 2 with overall expression levels were the SKAT-Burden and SKAT-O approaches.

### Limitations

For our simulation studies, we generated the rare variant data such that rare variants in the region acted in the same direction. If rare variants in the region were not directionally consistent, this could be an issue for the negative binomial regressions which collapsed rare variants in the region. However, this would not be an issue for the SKAT approaches, which further supports our recommendation.

### Future directions

We considered 2 approaches: (1) collapsing the rare variants in the region and using negative binomial regression or Poisson regression and (2) using the normalized read counts with SKAT, SKAT-O, or SKAT-Burden. Given that SKAT is based on generalized linear mixed models, another approach would be to extend SKAT for negative binomial traits.

## Supporting information

S1 File Supplementary simulation results. The file contains the results for all 60 plots of the 540 simulation scenarios considered (i.e. 9 fold changes for all combinations of *μ* = 50, 100, 500 and *n* = 15, 30, 50, 100, 500 subjects for scenarios A.1, A.2, B.1, B.2).
(PDF)
