# Supplementary material for: eQTL mapping of rare variant associations using RNA-seq data: An evaluation of approaches
Source: PLoS One. 2019 Oct 3;14(10):e0223273. doi: 10.1371/journal.pone.0223273 (PMC6776318; doi:10.1371/journal.pone.0223273)

Supplemental Figure 1: Scenario A.1: read counts are generated from a Poisson distribution with the average read count μ equal to 50 and the number of subjects equal to 15, 30, 50, 100, and 500, respectively.

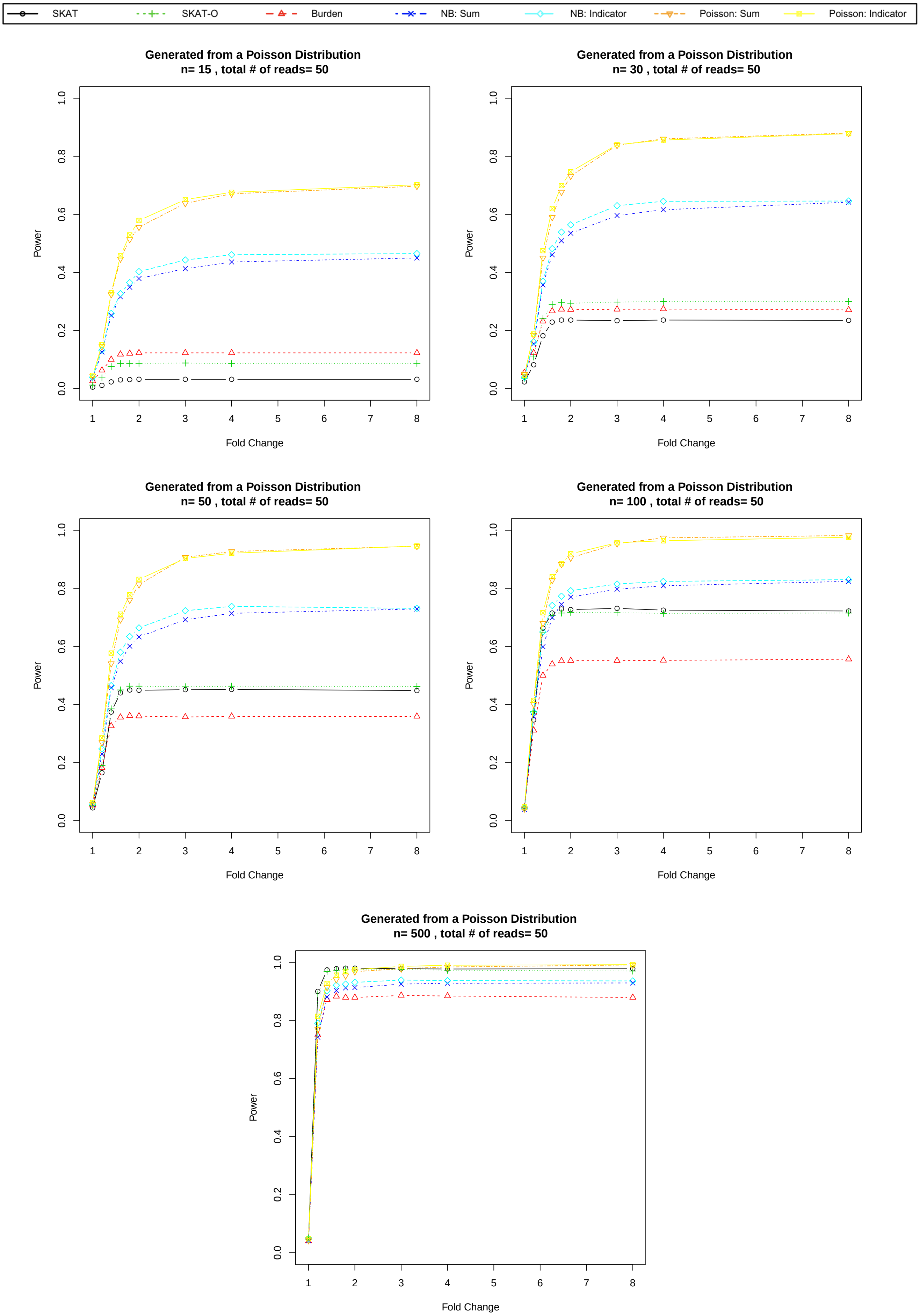

Supplemental Figure 2: Scenario A.1: read counts are generated from a Poisson distribution with the average read count μ equal to 100 and the number of subjects equal to 15, 30, 50, 100, and 500, respectively.

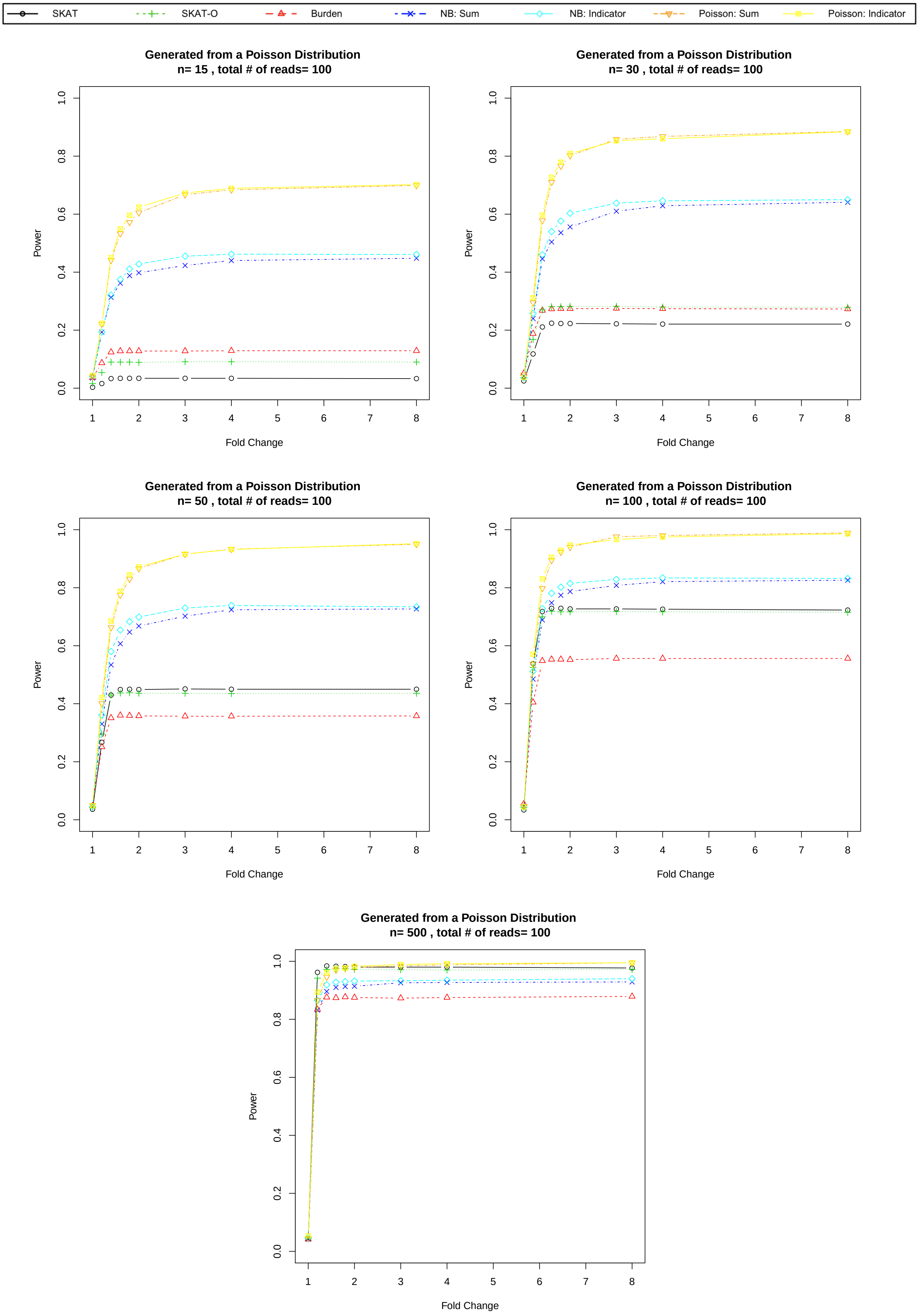

Supplemental Figure 3: Scenario A.1: read counts are generated from a Poisson distribution with the average read count μ equal to 500 and the number of subjects equal to 15, 30, 50, 100, and 500, respectively.

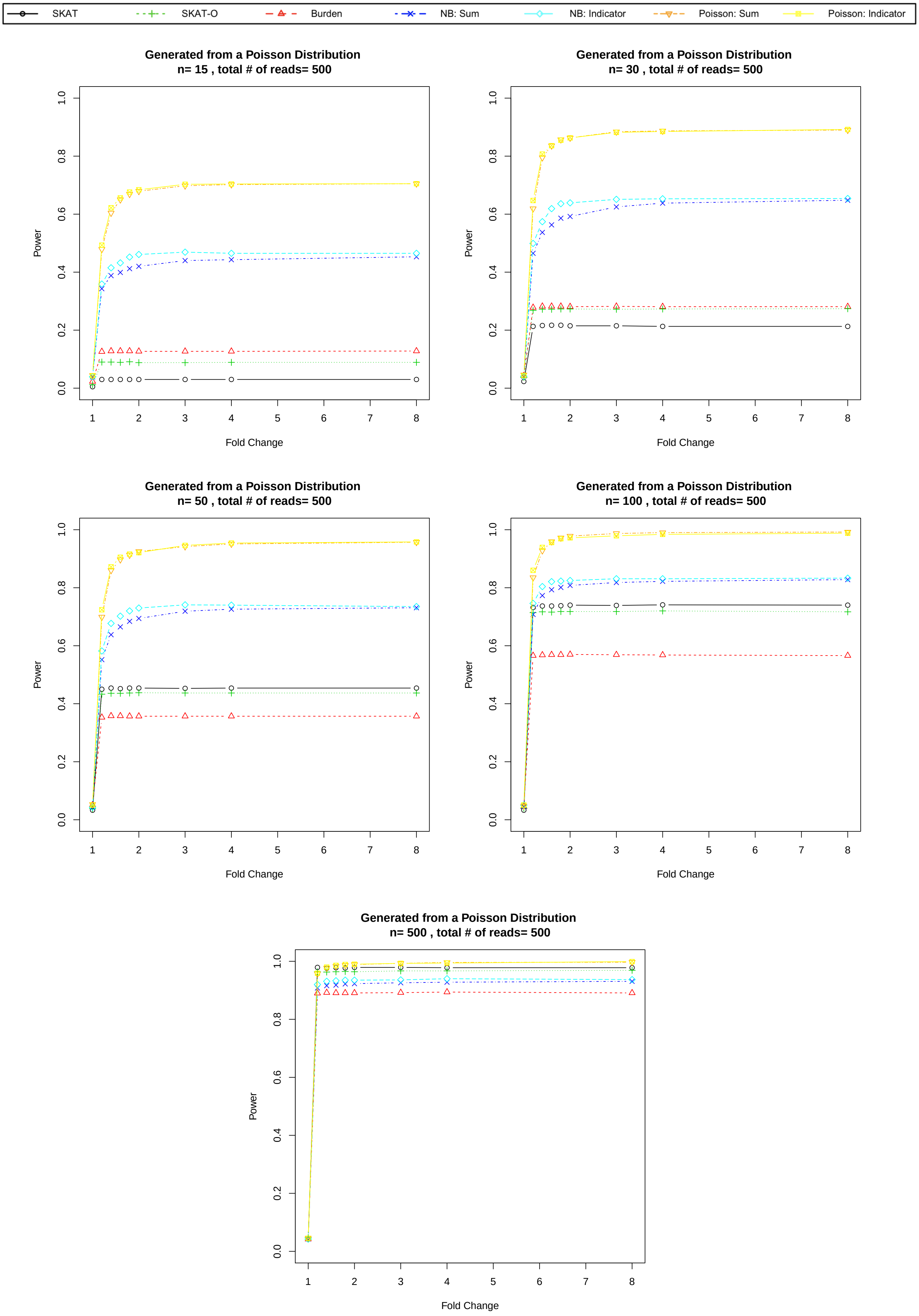

Supplemental Figure 4: Scenario A.2: read counts are generated from a negative binomial distribution with the average read count μ equal to 50 and the number of subjects equal to 15, 30, 50, 100, and 500, respectively.

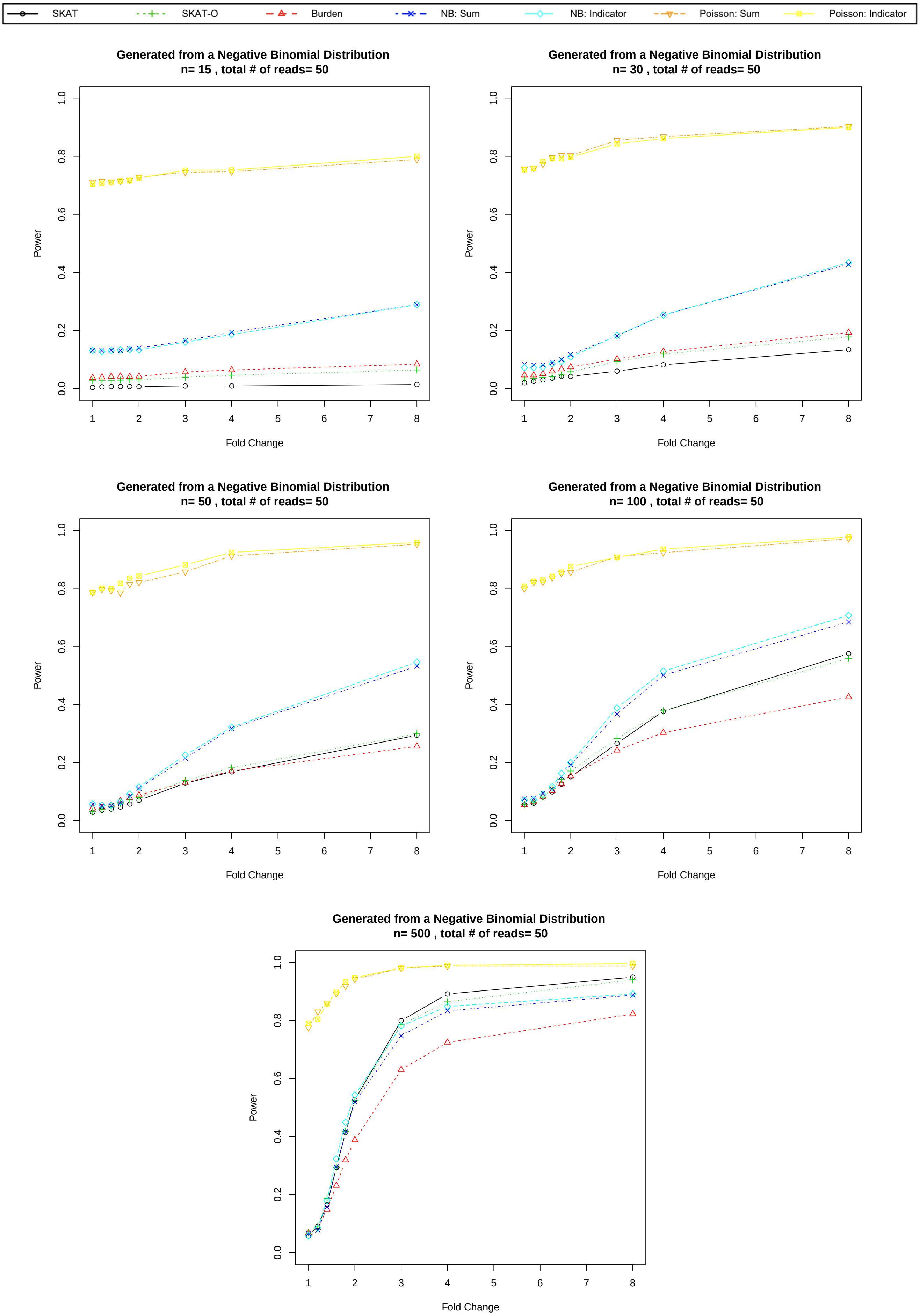

Supplemental Figure 5: Scenario A.2: read counts are generated from a negative binomial distribution with the average read count μ equal to 100 and the number of subjects equal to 15, 30, 50, 100, and 500, respectively.

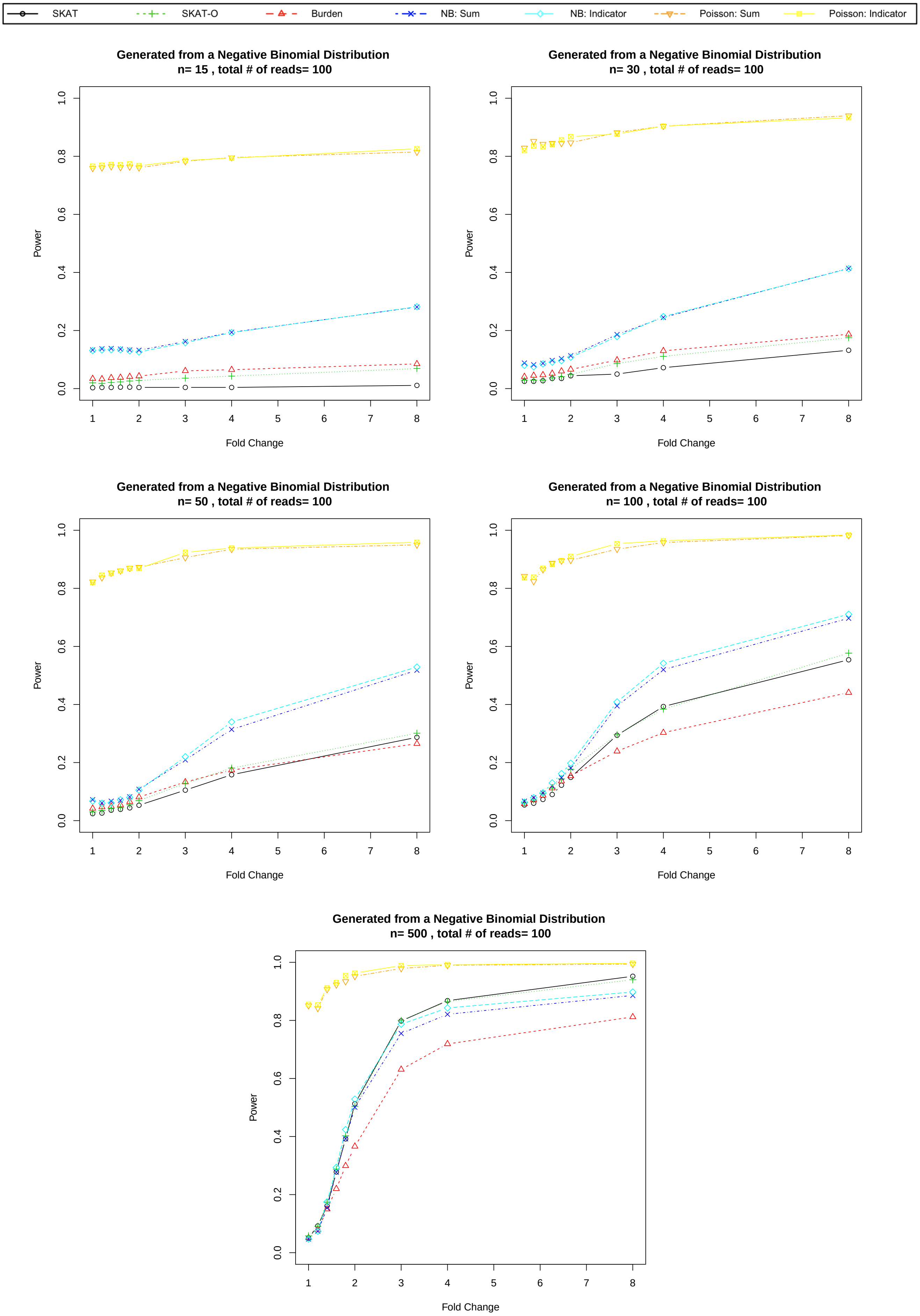

Supplemental Figure 6: Scenario A.2: read counts are generated from a negative binomial distribution with the average read count μ equal to 500 and the number of subjects equal to 15, 30, 50, 100, and 500, respectively.

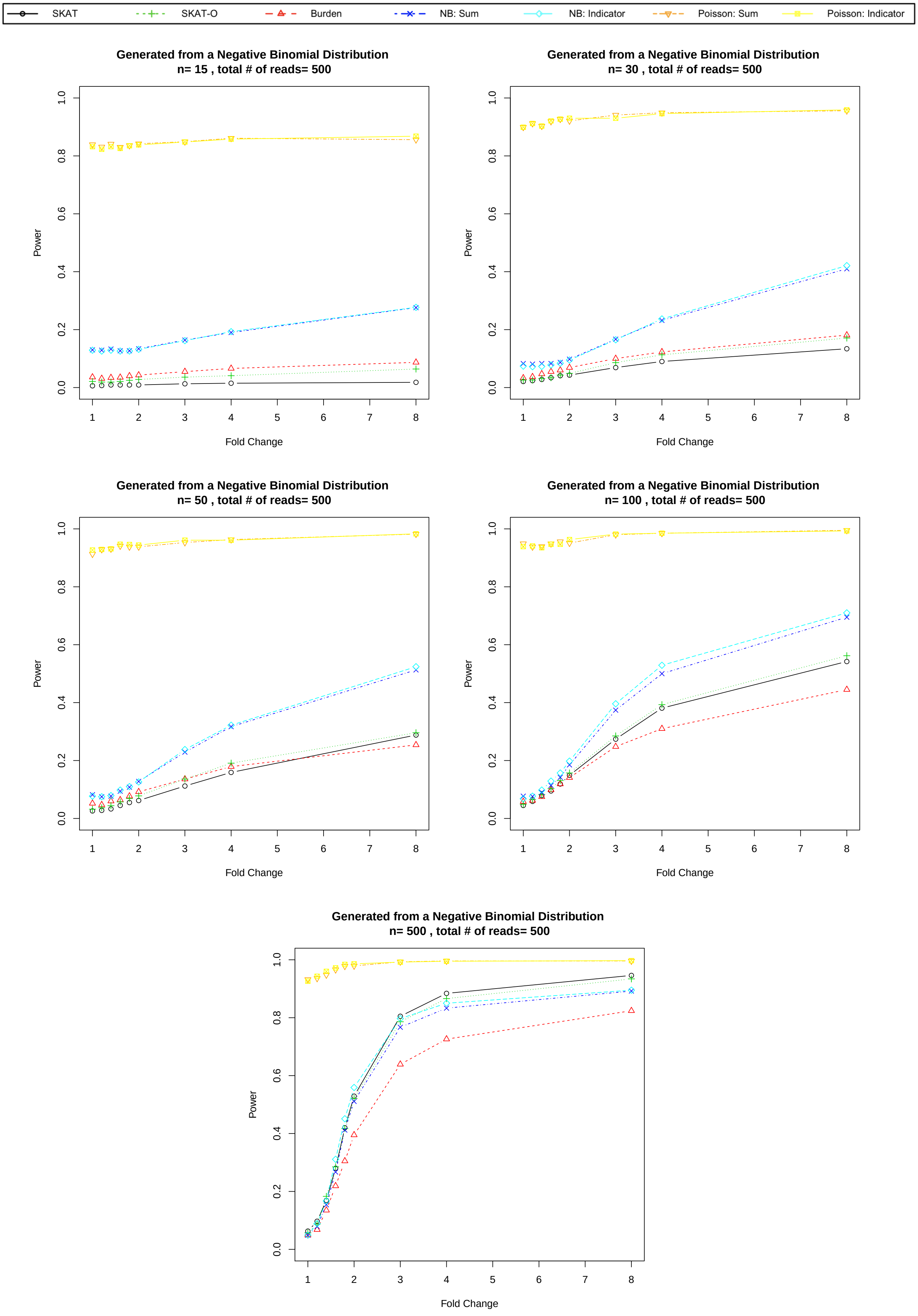

Supplemental Figure 7: Scenario B1: read counts are generated from a Poisson distribution with the average read count μ equal to 50 and the number of subjects equal to 15, 30, 50, 100, and 500, respectively.

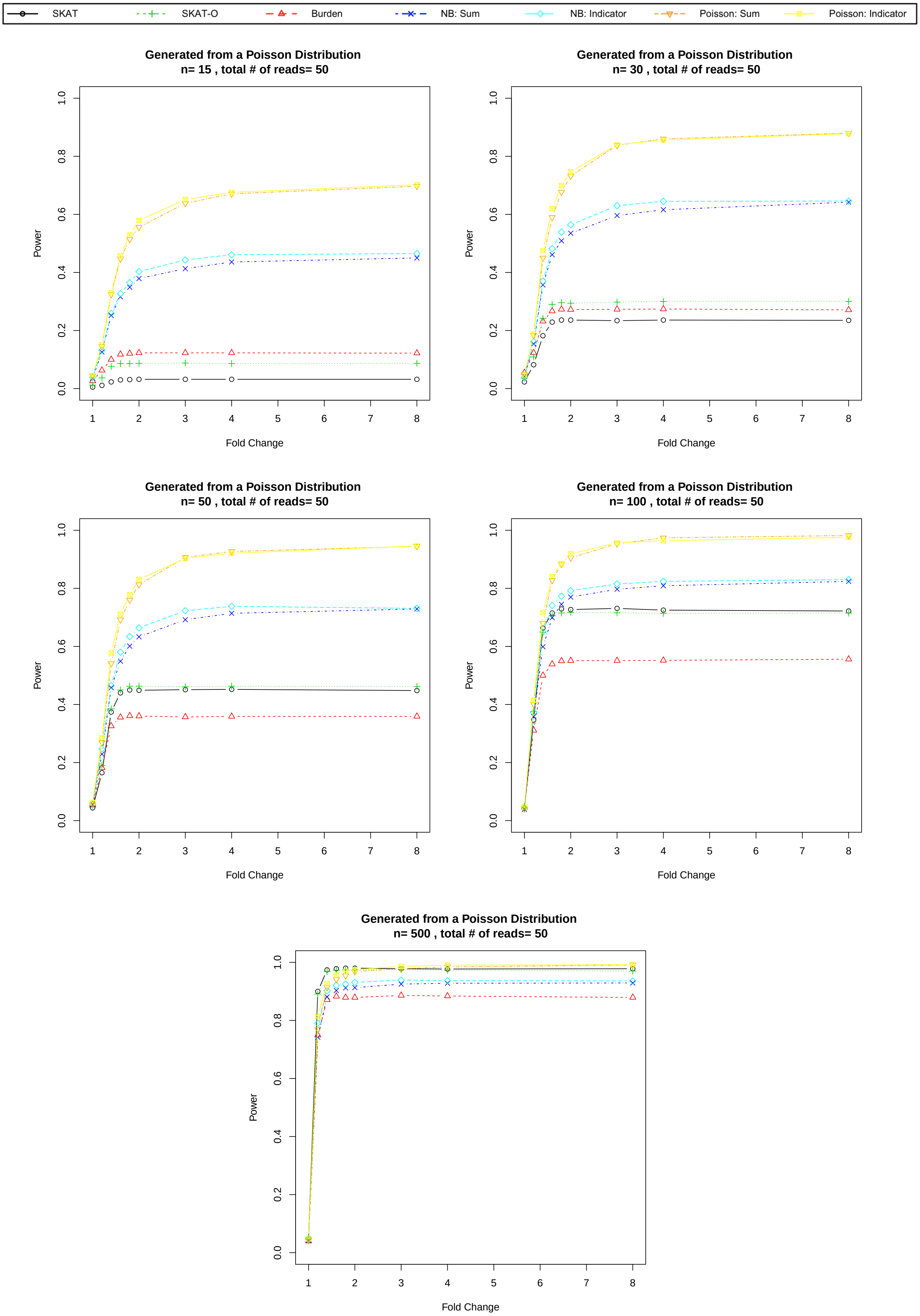

Supplemental Figure 8: Scenario B1: read counts are generated from a Poisson distribution with the average read count μ equal to 100 and the number of subjects equal to 15, 30, 50, 100, and 500, respectively.

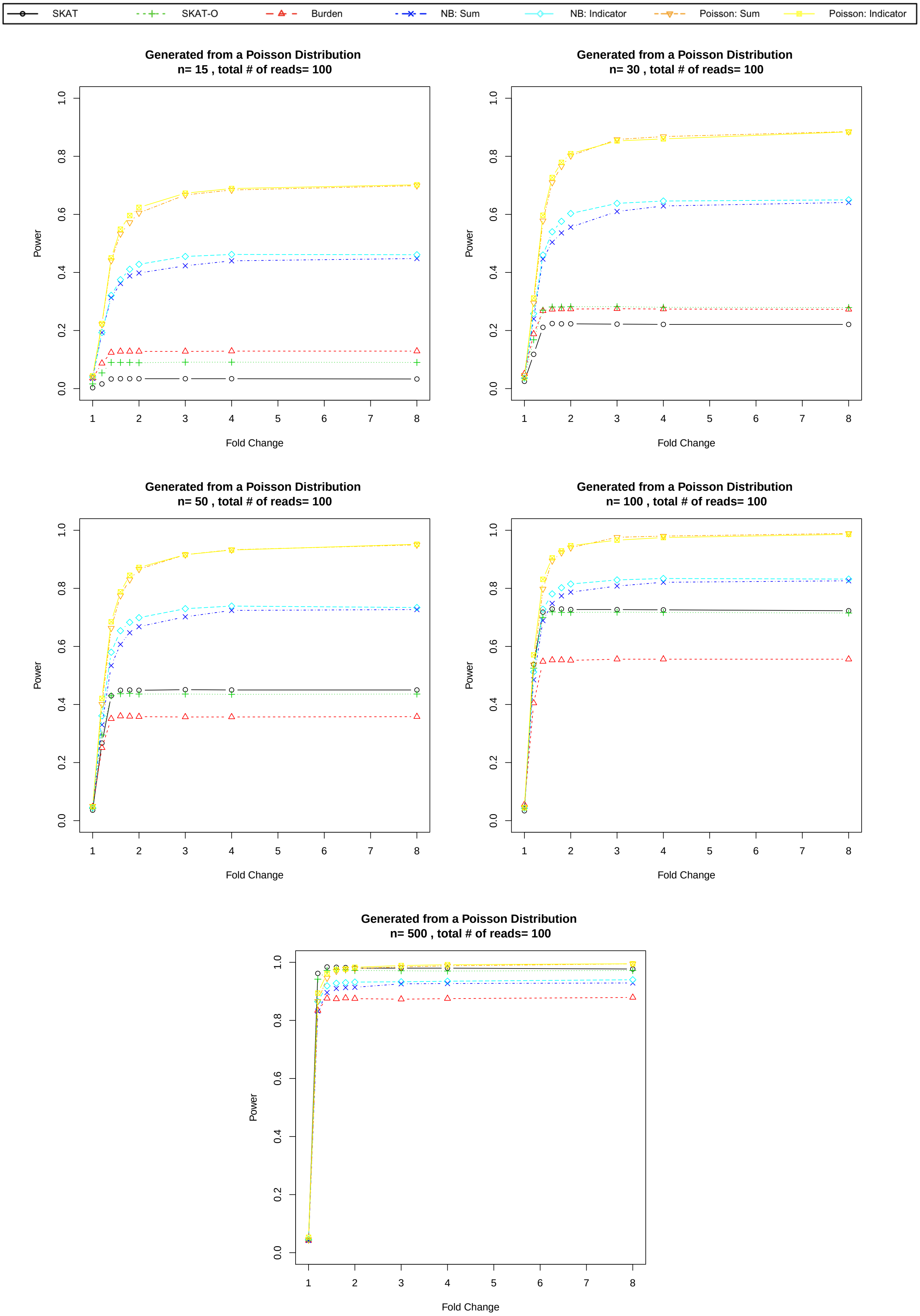

Supplemental Figure 9: Scenario B1: read counts are generated from a Poisson distribution with the average read count μ equal to 500 and the number of subjects equal to 15, 30, 50, 100, and 500, respectively.

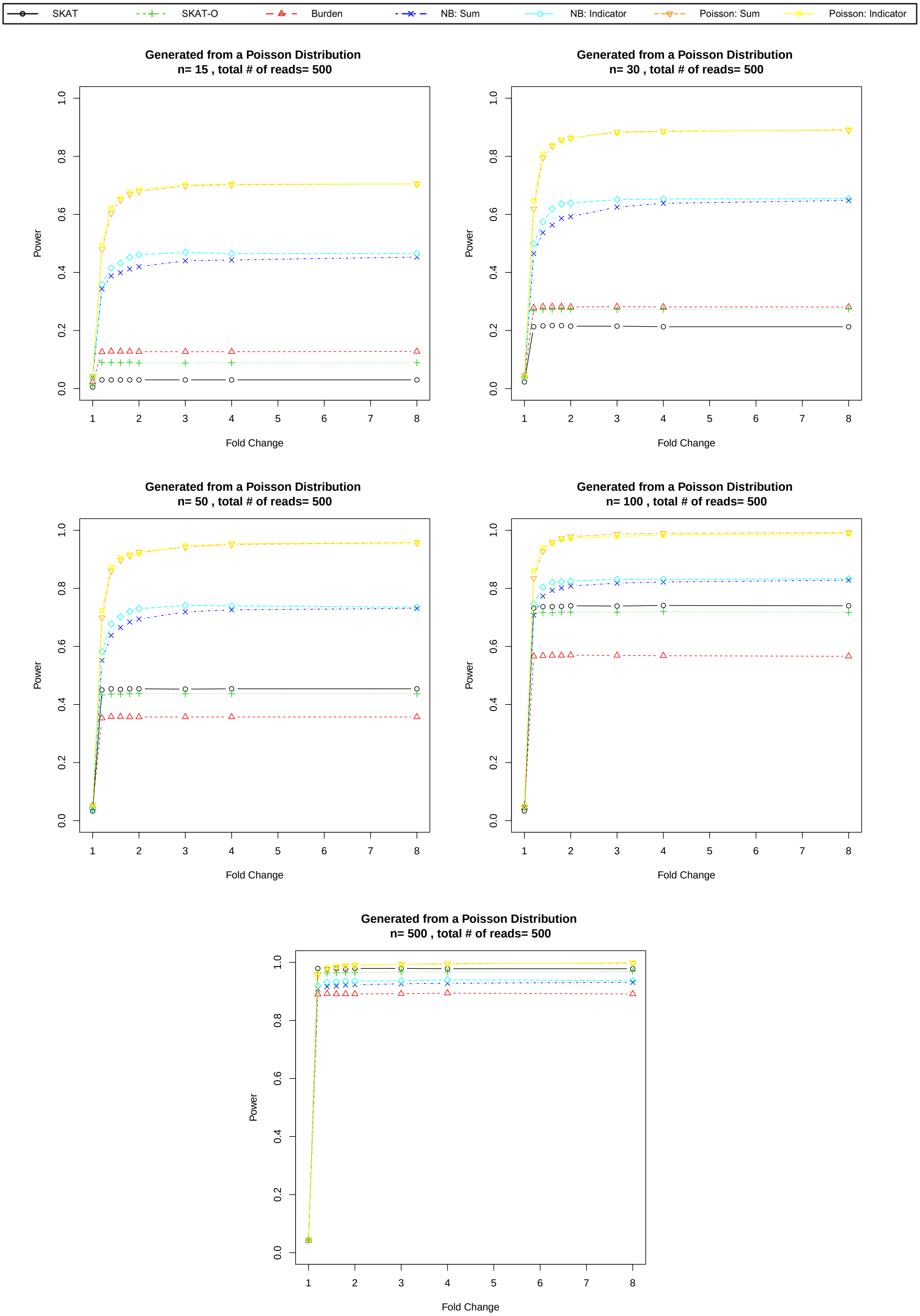

Supplemental Figure 10: Scenario B.2: read counts are generated from a negative binomial distribution with the average read count μ equal to 50 and the number of subjects equal to 15, 30, 50, 100, and 500, respectively.

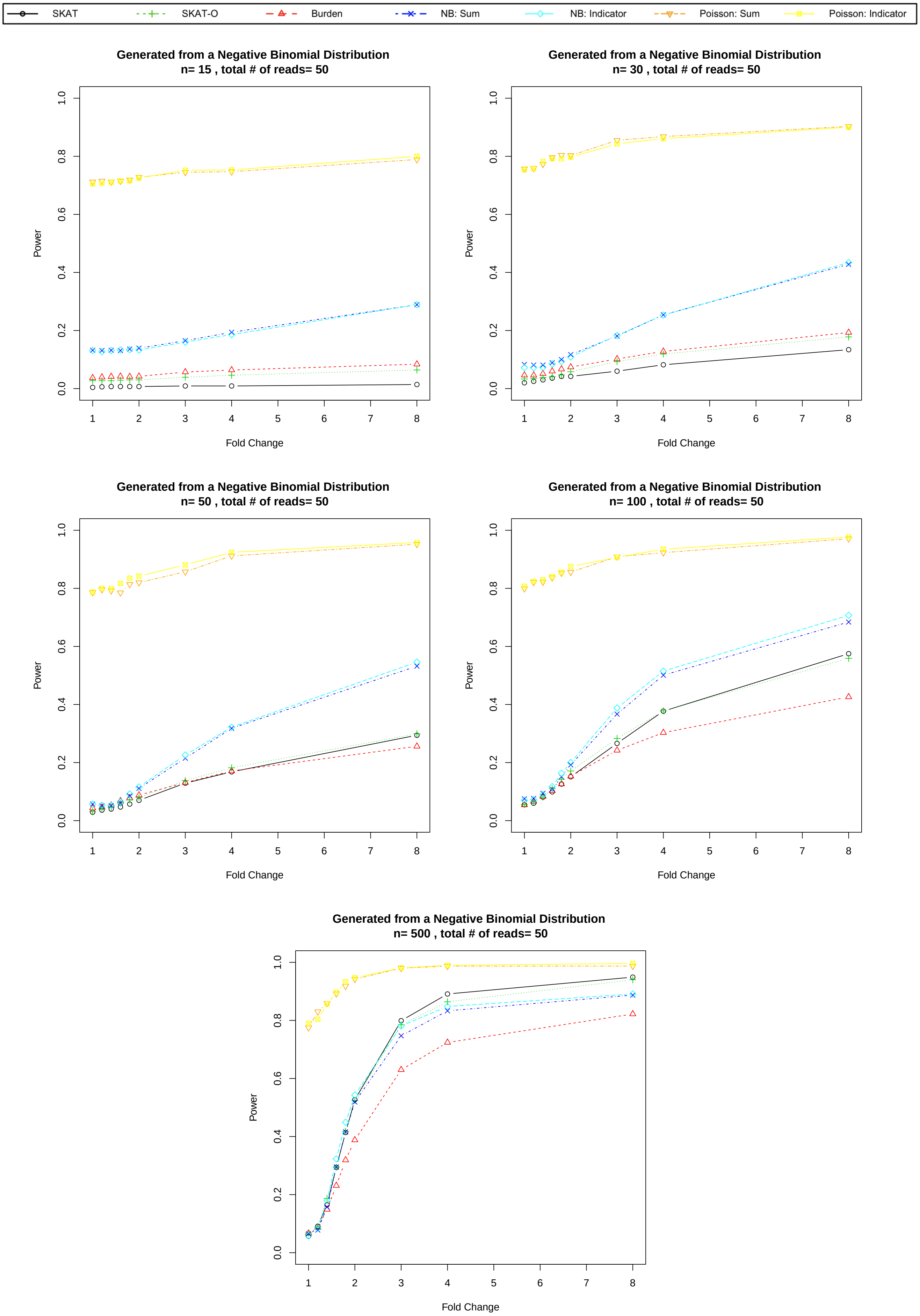

Supplemental Figure 11: Scenario B.2: read counts are generated from a negative binomial distribution with the average read count μ equal to 100 and the number of subjects equal to 15, 30, 50, 100, and 500, respectively.

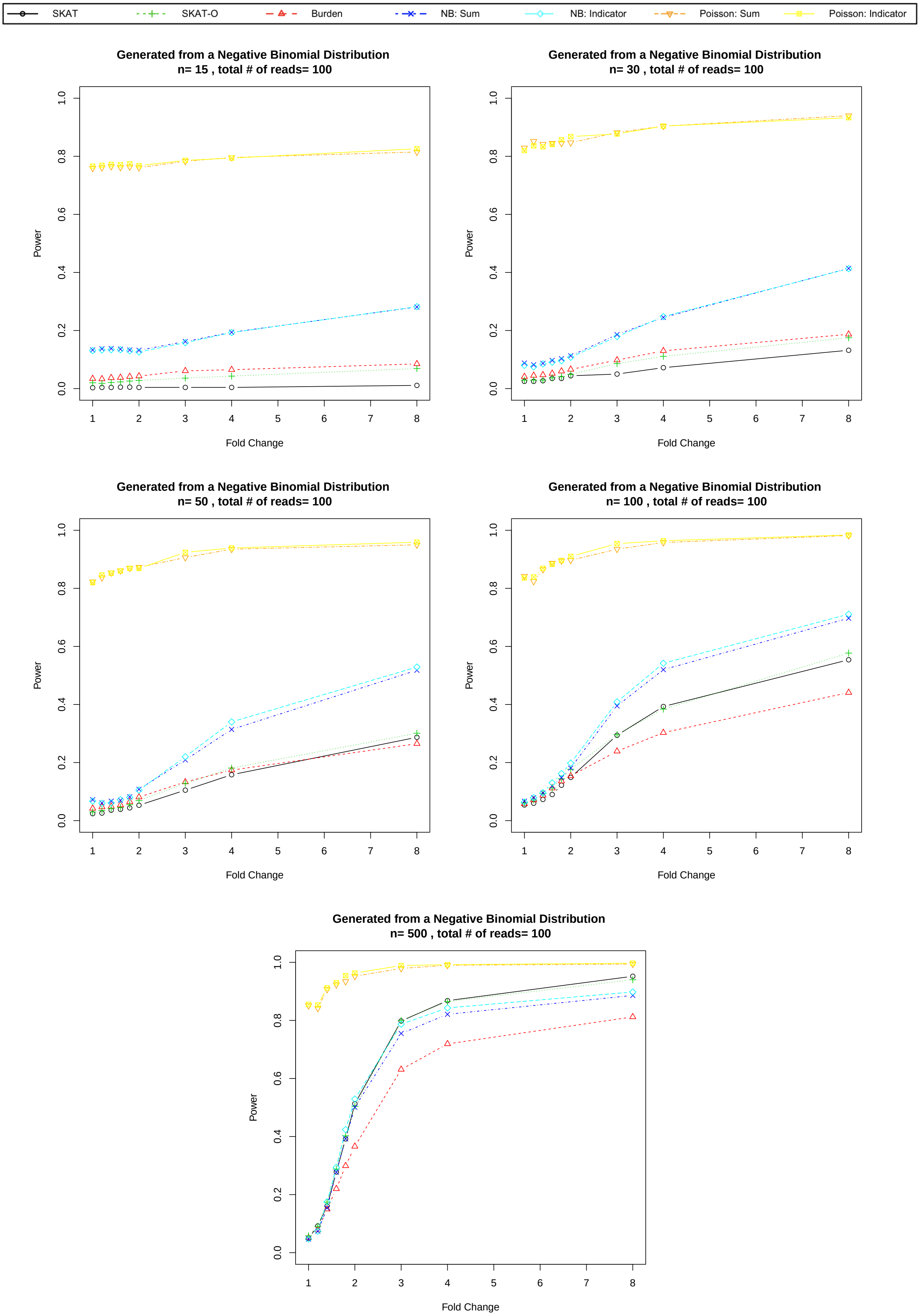

Supplemental Figure 12: Scenario B.2: read counts are generated from a negative binomial distribution with the average read count μ equal to 500 and the number of subjects equal to 15, 30, 50, 100, and 500, respectively.

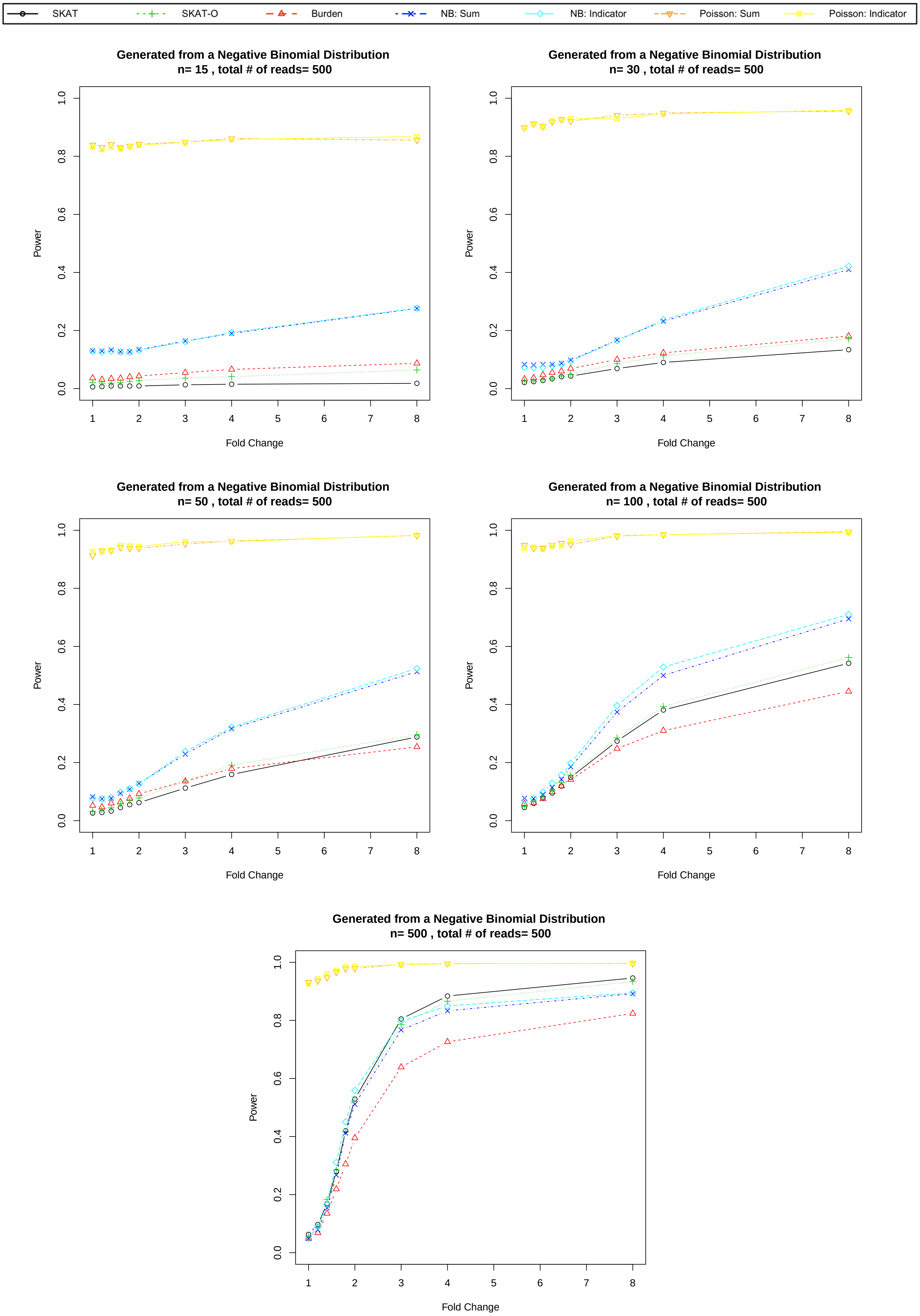

Supplement: S1 File — The file contains the results for all 60 plots of the 540 simulation scenarios considered (i.e. 9 fold changes for all combinations of μ = 50, 100, 500 and n = 15, 30, 50, 100, 500 subjects for scenarios A.1, A.2, B.1, B.2). (PDF) [file pone.0223273.s001.pdf]
